# Validity and reliability of ankle morphological measurements on computerized tomography-synthesized planar radiographs

**DOI:** 10.1186/s12938-016-0215-9

**Published:** 2016-08-05

**Authors:** Chien-Chung Kuo, Hsuan-Lun Lu, Tung-Wu Lu, Alberto Leardini, Mei-Ying Kuo, Horng-Chaung Hsu

**Affiliations:** 1 Institute of Biomedical Engineering, National Taiwan University, No. 1, Sec. 1, Jen-Ai Road, 100, Taipei, Taiwan, ROC; 2 Department of Orthopedics, China Medical University Hospital, Taichung, Taiwan, ROC; 3 Department of Orthopedic Surgery, School of Medicine, China Medical University, Taichung, Taiwan, ROC; 4 Department of Orthopedic Surgery, School of Medicine, National Taiwan University, Taipei, Taiwan, ROC; 5 Movement Analysis Laboratory, Istituto Orthopedic Rizzoli, Bologna, Italy; 6 Department of Physical Therapy, China Medical University, Taichung, Taiwan, ROC

**Keywords:** Reliability, Validity, Ankle morphology, Digitally reconstructed radiograph

## Abstract

**Background:**

Clinical success of total ankle arthroplasty depends heavily on the available information on the morphology of the bones, often obtained from measurements on planar radiographs. The current study aimed to evaluate the intra-rater, inter-rater and inter-session reliability and the validity of radiograph-based measurements of ankle morphology, and to quantify the effects of examiner experience on these measurements.

**Methods:**

Twenty-four fresh frozen ankle specimens were CT scanned, data of which were used to reconstruct 3D volumetric bone models for synthesizing 2D radiographs. Two orthopaedic surgeons with different levels of clinical experience identified twenty landmarks five times on each of the synthesized sagittal and coronal radiographs and repeated the test on a subsequent day within 5 days. The landmarks were used to calculate fourteen morphological parameters. The two-way mixed-effects (ICC3,1), two-way random-effects (ICC2,k) and two-way random-effects (ICC3,k) models were used, respectively, to assess the intra-rater, inter-rater and inter-session reliability of measurements. The validity of the measurements for each examiner was assessed by comparing them with gold standard values obtained from the 2D radiographs projected from the 3D volumetric models using Pearson’s correlation analysis and Bland and Altman plots, and the differences were defined as the measurement errors.

**Results:**

Most of the morphological parameters were of good to very good intra-rater, inter-session and inter-rater reliability for both examiners (ICC > 0.61). Experience appeared to affect the inter-rater and inter-session reliability, the senior examiner showing greater inter-session ICC values than the junior examiner. Most of the tibial parameters had moderate to excellent correlations with the corresponding gold standard values but were underestimated by both examiners, in contrast to most of the talar parameters that were overestimated and had only poor to fair correlations.

**Conclusions:**

Most of the morphological parameters of the ankle can be estimated from radiographs with good to very good intra-rater, inter-session and inter-rater reliability, for both clinically experienced and less experienced examiners. Clinical experience helped increase the reliability of repeated evaluations after a longer interval, such as in a follow-up assessment. It is suggested that critical clinical decisions based on repeated morphology measurements should be made by more experienced surgeons or after appropriate training.

## Background

Arthritis of the ankle joint often leads to impairment of locomotion, physical disability and reduced quality of life [1–5]. Ankle arthrodesis is effective for pain relief and in restoring joint stability, but sacrifices joint mobility, which can seriously affect locomotion [6–8]. Total ankle arthroplasty (TAA) is an important alternative to arthrodesis [4, 9–11], especially for the management of advanced ankle osteoarthritis (OA), because it not only relieves pain and restores joint stability, but it also restores mobility of the joint [12]. Although increased complications and high failure rates of TAA as compared to arthrodesis have led many surgeons to choose arthrodesis for treating ankle arthritis [13], more modern prosthesis designs have contributed to a renewed interest in TAA over the past decade [14]. Clinical success of TAA depends heavily on the available information on the morphology of the relevant bones [15], which is critical for the design of ankle prostheses and for the procedures of their surgical implantation [1, 2]. It has been suggested that restoration of the ankle joint using TAA based on anatomical dimensions leads to the best clinical results [6–8]. Current advancements in manufacturing will lead to personalized solutions for human joint replacements [16], which necessarily must be based on accurate morphological measurements of the individual patients [17, 18].

The conformity of the TAA design to the bone morphology, including proper sizing of the components, is an important factor for the prosthesis to replicate the function of the ankle joint [8]. Using implants of sizes and shapes precisely matching the osteotomies is expected to be of value for the long-term fixation of the implants [13, 19, 20], and can substantially reduce complications and increase survival rates [12–14]. Therefore, errors in the measurement of the patient-specific morphological parameters may have critical effects on the pre-surgical decision-making in TAA, including the selection of the size of the implants.

Among the clinically available medical imaging modalities, planar radiographs are commonly used in standard clinical practice. These can also be used for estimating patient-specific morphological parameters and for selecting the size of the prosthesis, particularly owing to their convenience, low cost, and low radiation dose compared with other modalities such as Magnetic Resonance Imaging or Computerized Tomography (CT) [21]. However, since planar radiographs are two-dimensional (2D) projective images, bones at different distances from the projection plane produce bone images of different size, position and intensity, critically affecting the measurement accuracy [19]. In addition, the intrinsic articular surfaces of the ankle joint are not symmetrical, and are often oblique with respect to the anatomical planes of the shank and foot. It is thus difficult to obtain accurate patient-specific morphological parameters using single planar radiographs, either in the anteroposterior (A/P), mediolateral (M/L) or mortise views, leading also to errors in image interpretation. The difficulty is further increased when the ankle is affected by OA with spur formation, joint mal-alignment or trauma with broken bone contours. With the original anatomy altered by injury or diseases, the accuracy of the manual identification of relevant bony landmarks necessary for measuring morphological parameters is critically affected, resulting in larger measurement errors, leading to uncertainties in the clinical decision-making and in the planning and evaluation of treatment, as well as in the selection of a suitable total ankle replacement. Moreover, since these morphological measurements may be taken by the same or by different clinicians with different levels of experience, and at different stages in the management of the patient, it is necessary to determine whether the measurements used are valid, as well as reliable, both within (intra-rater) and between clinicians (inter-rater), and between sessions (inter-session).

Studies on the validity and reliability of ankle morphological measurements on planar radiographs have been limited. Murphy et al. [20] produced the only study on the reliability of measuring the medial and superior clear spaces of the normal ankle on planar radiographs in terms of ICC values. To the best of our knowledge, no studies have evaluated quantitatively the validity and reliability of a more complete range of morphological measurements on planar radiographs. A major difficulty is the concurrent definition of the three-dimensional (3D) gold standard while generating planar radiographs for repeated 2D measurements either in vivo or in vitro. For example, markers placed on the bony landmarks for 3D measurements will also appear on the planar radiographs, which will affect the subsequent independent repeated measurements on the radiographs. By taking advantage of CT-based computer simulation, gold standard values can be established by 3D CT measurements and the repeated planar measurements can be made on 2D radiographs concurrently synthesized using the CT data [19]. Comparisons between known gold standards based on 3D data and corresponding 2D repeated measurements will help assess quantitatively the validity and reliability of standard ankle morphology measurements based on routine planar images.

The purpose of this study was to evaluate the validity and inter-rater, intra-rater, and inter-session reliability of radiograph-based measurements of ankle morphology, and to quantify the effects of examiner experience on these measurements.

## Methods

### Specimen preparation

**Fig. 1:**
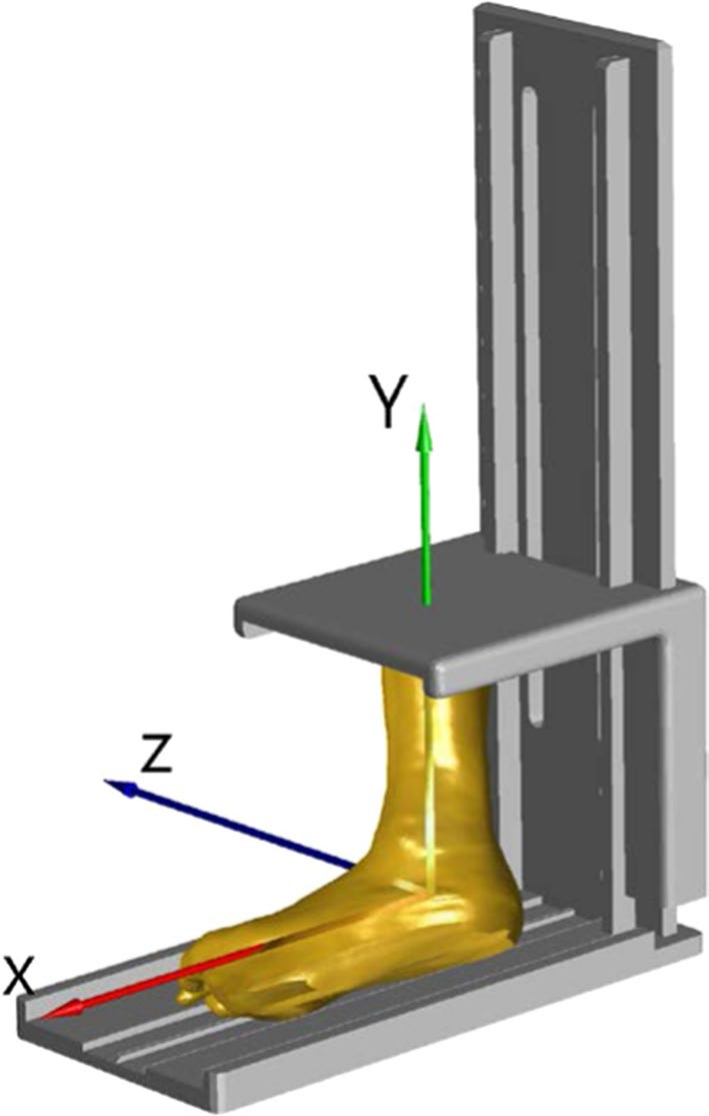
Positioning of the ankle specimen and definition of the coordinate system

Twenty-four fresh frozen ankle specimens (Table 1) were used. These were obtained from donors who had undergone below-knee amputation procedures for reasons other than trauma or disease of the ankle joint. The specimens were stored at −70 °C immediately after harvest and thawed at room temperature 24 h prior to experiments. Each ankle specimen was positioned in the neutral position in a plastic frame (Fig. 1) according to previously determined procedures [22]. The specimen was fixed to a base-plate using bone cement, with the long axis of the base-plate of the frame aligned with the line joining the calcaneal insertion of the Achilles tendon and the second metatarsal head. The neutral position of the ankle specimens was then defined when the longitudinal axis of the shank was perpendicular to the base-plate as indicated by a goniometer. The spine of the plastic frame was adjusted to accommodate specimens with different lengths of the remaining part of the shank. The proximal ends of tibia and fibula were fixed to the upper plate using bone cement. This procedure enabled a reliable definition of the anatomical frame of reference for the specimen as a whole [22]. After specimen fixation, the entire construct was scanned with a 16-slice spiral CT scanner (GE BrightSpeed16, C&G Technologies, USA) with a slice thickness of 0.625 mm. The resolution of the obtained CT images was 512 × 512 (pixels) and the voxel size was 0.630 × 0.630 × 0.625 (mm^3^).

**Table 1:** Demographic data of the donors of the ankle specimens

	All (n = 24)		Male (n = 12)		Female (n = 12)	
	Mean (SD)	[Min max]	Mean (SD)	[Min max]	Mean (SD)	[Min max]
Age (years)	66.3 (12.2)	[40 87]	63.1 (11.8)	[40 83]	70.8 (11.7)	[51 87]
Height (cm)	162.4 (7.1)	[150 175]	164.4 (6.9)	[152 175]	159.5 (6.7)	[150 170]
Body mass (kg)	64.6 (12.2)	[46 95]	63.8 (8.6)	[48 81]	65.7 (16.2)	[46 95]

### CT-based bone models and morphological parameters

**Fig. 2:**
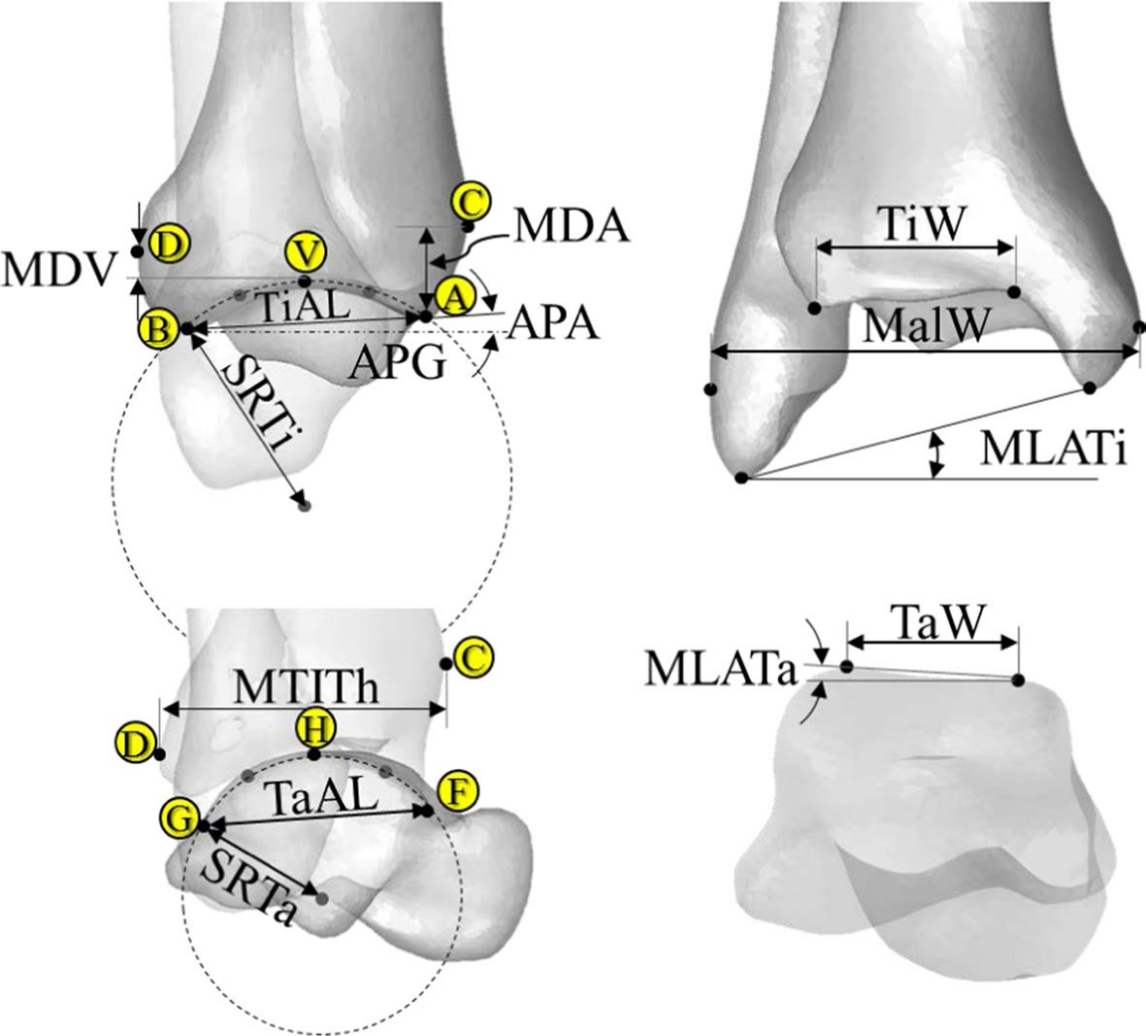
Graphical depiction of the ankle morphological parameters. The ankle morphological parameters are defined on the 3D bone models as seen in the sagittal (a–b) and frontal (c–d) planes. Letters in *yellow circles* identify relevant landmarks (see also Table 2 for all these definitions)

Given the CT images for each specimen, the 3D volumetric models of the tibia, fibula and talus bones together with the plastic frame were reconstructed using a commercial software package (Amira 3.2, VSG, USA). An anatomical reference coordinate system was embedded in the construct for subsequent quantitative descriptions of ankle morphology (Figs. 1, 2). The origin of this coordinate system was taken to be at the geometric center of the talus. The anteroposterior (A/P) axis was defined as the line joining the calcaneal insertion of the Achilles tendon and the head of the second metatarsal, and was orientated parallel to the base-plate. The superoinferior (S/I) axis was taken as the vector perpendicular to the base-plate, which closely followed the longitudinal axis of the shank that was carefully positioned during specimen fixation, guided by a goniometer. The mediolateral (M/L) axis was then defined as the line perpendicular to both the A/P and S/I axes. The anatomical landmarks required for the definition of the morphological parameters were identified automatically for each of the bone models based on the 3D geometrical definitions (Table 2; Fig. 2) [10, 22] using an in-house written program in MATLAB (R2010a, The MathWorks, Inc., USA). The procedure was previously shown to have high reliability and accuracy [22].

**Table 2:** Definitions of the parameters used to describe the morphology of the ankle joint

Distal tibia	
TiAL (mm)	*Tibial arc length* distance between the most anterior (A) and posterior (B) points of the maximal arc of the tibial mortise in the sagittal plane
TiSR (mm)	*Tibial sagittal radius* radius of the AB arc
APG (mm)	*Antero*-*posterior gap* supero-inferior component of the distance between A and B
APA (deg)	*Antero*-*posterior inclination angle* inclination angle between the antero-posterior axis and the AB segment
MTiTh (mm)	*Maximal tibial thickness* the A/P distance from the most anterior (C) to the most posterior (D) point on the tibial profile in the sagittal plane
MDA (mm)	Supero-inferior distance between A and C
MDV (mm)	Supero-inferior distance between the most proximal vertex of the tibial mortise (V) and the point D
TiW (mm)	*Tibial width* medio-lateral distance of the tibial mortise calculated using the two end-points of the anterior and posterior edges
MalW (mm)	*Malleolar width* medio-lateral distance between the most lateral point of the fibula and the most medial point of the tibia
MLATi (deg)	Angle in the frontal plane between the medio-lateral axis and the line joining the most distal points of the fibula and tibia
Talus	
TaAL (mm)	*Trochlea tali arc length* distance between the most anterior (F) and posterior (G) and proximal (H) points of the trochlea tali, as seen in the sagittal projection of the talus
TaW (mm)	*Trochlea tali width* width between medial and lateral crests of the talar dome
TaR (mm)	*Trochlea tali radius* radius of the talar dome in the sagittal plane, as identified by the arc FG
MLATa (deg)	Angle in the frontal plane between the medio-lateral axis and the line joining the two most proximal vertices of the trochlea tali

See Fig. 2 for graphical descriptions

### Generation of digitally reconstructed radiograph (DRR)

**Fig. 3:**
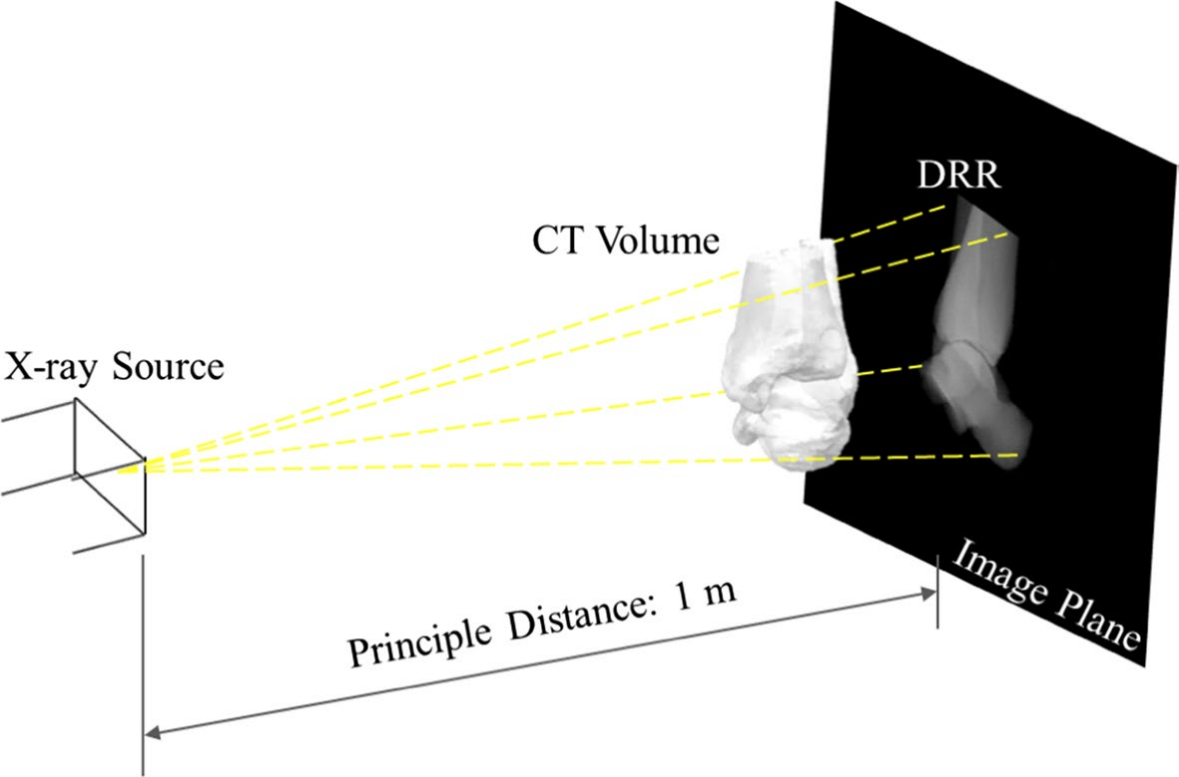
Generation of digitally reconstructed radiograph (DRR). Diagram for the generation of DRR of the ankle joint in the neutral position using a perspective projection of the CT data of the ankle specimen

For repeated 2D measurements, 2D radiographs were synthesized from the 3D CT-based bone models using the technique of digitally reconstructed radiographs [19]. Given the positions of the X-ray source and a CT-derived volumetric ankle model in space with respect to the image plane, the DRR was generated by casting rays through the volume of the CT-based volumetric ankle model [21]. Each of these rays went through a number of voxels of the volume, the attenuation coefficients of which were then integrated along the ray and projected onto the imaging plane to obtain a DRR image resembling a radiograph (Fig. 3). In order to reduce the time required for DRR generation, the ray-tracing was implemented with trilinear interpolation in MATLAB [23]. The DRRs were generated simulating the standard X-ray imaging of the ankle on a digital radiography system (CXDI-40EG, CANON, USA) in which the X-ray focus was 1 m away from the image plane. The most lateral point of the ankle model, i.e., the most lateral projection of the lateral malleolus, was in contact with the image plane for M/L imaging, and the most posterior point, i.e., the most posterior projection of the calcaneus, was in contact with the image plane for A/P imaging (Fig. 3) [21]. The target of the X-ray was set at the medial malleolus for M/L imaging, and at the mid-point between the two malleoli for A/P imaging. Standard sagittal (M/L) and frontal (A/P) DRR-synthesized radiographs were created from the CT data for each specimen for subsequent manual morphological measurements. For the definition of the gold standard values for planar measurements, the landmarks on the bone models were also projected onto the image plane, which enabled the automatic calculation of the gold standard values of a total of fourteen morphological parameters, nine for the tibia-fibula segment and five for the talus [19, 22], as based on the definitions given in Fig. 2 and Table 2, and using an in-house developed program in MATLAB.

### Measurement protocol

**Fig. 4:**
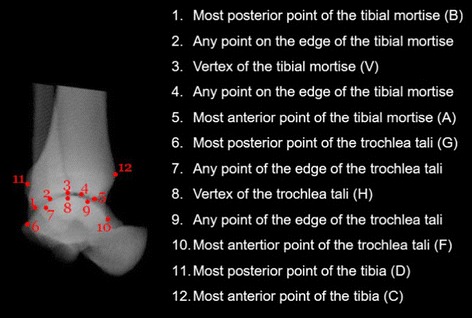
Mediolateral DRR of the ankle with twelve bony landmarks identified. Illustration of the sequence of identification of the 12 bony landmarks on the M/L DRR. The *numbers* indicate the sequence of the landmarks to be identified by the examiner. For each landmark, a brief description is given. Detailed definitions of the landmarks (some denoted by *Latin letters*) are given in Fig. 2 and Table 2

**Fig. 5:**
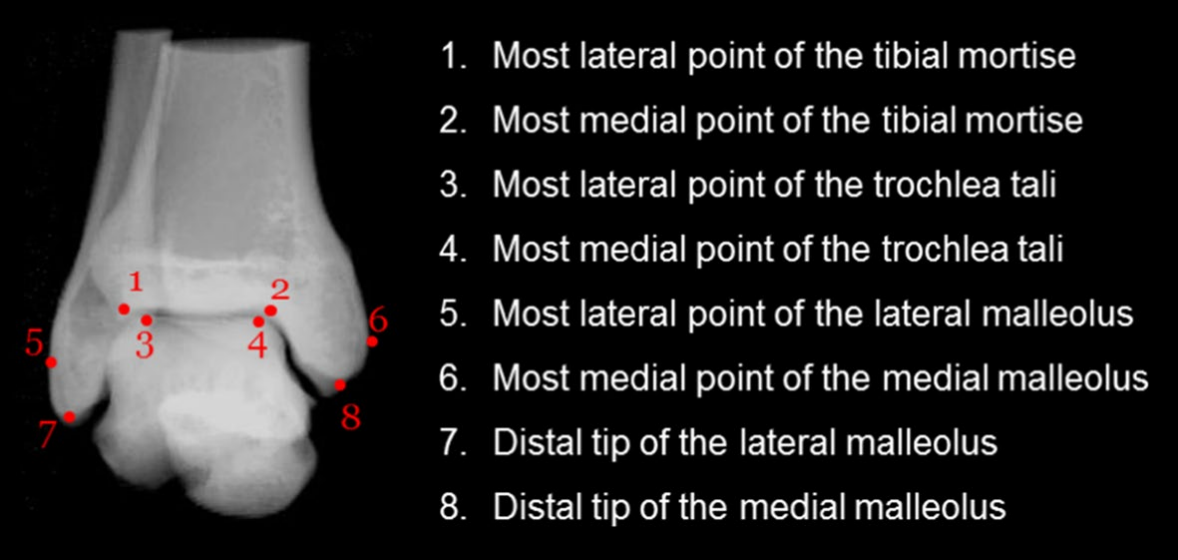
Anteroposterior DRR of the ankle with eight bony landmarks identified. Illustration of the sequence of identification of the 12 bony landmarks on the A/P DRR. The *numbers* indicate the sequence of the landmarks to be identified by the examiner. For each landmark, a brief description is given. Detailed definitions of the landmarks are given in Fig. 2 and Table 2

Two orthopaedic surgeons, one with 16 years of experience and the other with 2 years, participated in the current study as examiners. They were asked to identify the twenty bony landmarks necessary for defining morphological parameters on each of the synthesized sagittal and coronal radiographs (Figs. 4, 5) using the mouse pointer and with the assistance of a graphics-based user interface on a personal computer. Before the experiment the examiners were allowed to practise using the software for 10 min with radiographs not included in the current study. For each image, this procedure was repeated five times. The re-test was performed at approximately the same time of the day on a following day within a period of 5 days after the first session, following exactly the same test procedure.

### Calculation of the 2D morphological parameters

On the DRR-synthesized M/L radiograph, parameters describing the tibial morphology on the sagittal plane were tibial arc length (TiAL), tibial sagittal radius (TiSR), anterio-posterior gap (APG), anterio-posterior inclination angle (APA), maximal tibial thickness (MTiTh), anterior tibial thickness (MDA), tibial plafond thickness (MDV), and the ratio between the distances of AV and BV (TiPD) (Fig. 2; Table 2). Similarly, parameters describing the trochlea tali were its arc length (TaAL) and its radius (TaR) (Table 2). On the DRR-synthesized A/P radiograph, parameters for the morphology of the tibia were the tibial (TiW) and malleolar (MalW) widths, and the angle between the M/L axis and the line joining the most distal points of the tibia and fibula (MLATi) (Table 2). Similarly, parameters selected for the talar interface were the trochlea tali width (TaW), and the angle between the M/L axis and the line joining the two most proximal vertices of the trochlea tali (MLATa) (Table 2; Fig. 2).

### Data analysis

The values of each of the morphological parameters were ensemble-averaged across all specimens for each examiner, giving means, standard deviations (SD) and coefficients of variance (CV). Reliability between measures was assessed in terms of the intraclass correlation coefficient (ICC) [24] using a two-way mixed-effects model (ICC_3,1_) for intra-rater assessments and a two-way random-effects model (ICC_2,k_) for inter-rater assessments. For analysis of intra-session reliability, a two-way mixed-effects model (ICC_3,1_) was used while a two-way random-effects model (ICC_3,k_) was used for inter-session reliability. Values of the ICC ranging from 0.81 to 1.0 indicated very good reliability; 0.61–0.80 good; 0.41–0.60 moderate; 0.21–0.40 fair; and below 0.2 poor reliability [25].

The validity for each examiner was assessed by comparing the measurements for each morphological parameter with corresponding measurements on the projected gold standard images, their relationship being assessed using Pearson’s correlation analysis, and the differences defined as the measurement errors. For each examiner, Pearson’s correlation coefficients for each morphological parameter were calculated using the averaged values measured over the five repetitions. A correlation coefficient larger than 0.75 was defined as high to excellent correlation; 0.50–0.75 as moderate correlation, 0.25–0.5 as fair correlation, and 0.00–0.25 as poor or no correlation [24]. Paired *t* tests were performed to compare differences between the 2D measurements and the gold standard values for each examiner, and the associated effect sizes (Cohen’s *d*) were also obtained. Generally, an effect size of 0.8 was defined as large, 0.5 as medium, and 0.2–0.3 as small [24]. The bigger the effect size, the stronger the relationship between measurements and gold standard values would be. All significance levels were set at α = 0.05. Bland and Altman plots [25] were used to visualize the difference between 2D measurements by each examiner and gold standard values against the corresponding mean of the two sets of data for each subject with the bias (mean difference) and the 95 % confidence intervals of the bias indicated on the plots [25]. All statistical analyses were performed using a statistical software package (SPSS v.13; SPSS Inc., Chicago, IL, USA).

## Results

More than half of the estimated parameters showed significant differences between the two examiners (Table 3). However, good to very good intra-rater reliability was found in most of these parameters for both examiners, except for moderate reliability found for SRTa by both examiners and SRTi by the junior examiner, and for poor reliability for MLATa by both examiners (Table 3).

**Table 3:** Means (standard deviations, SD) of the ankle morphological parameters and the intra-rater reliability of measurements in terms of intra-class correlation coefficients (ICC) by the senior and junior examiners (n = 24)

	Senior		Junior		*p* value
	Mean (SD)	ICC	Mean (SD)	ICC	
TiAL (mm)	27.50 (2.67)	0.82	26.84 (2.28)	0.81	0.03
SRTi (mm)	26.74 (5.94)	0.75	27.24 (6.07)	0.55	0.64
APG (mm)	4.90 (3.75)	0.97	4.62 (3.49)	0.96	0.22
APA (deg)	10.10 (7.31)	0.96	9.88 (7.18)	0.95	0.61
MTiTh (mm)	42.25 (2.80)	0.88	40.30 (3.04)	0.87	0.00
MDA (mm)	6.76 (4.13)	0.92	5.30 (3.47)	0.87	0.04
MDV (mm)	9.28 (3.14)	0.88	7.33 (4.19)	0.93	0.00
TiW (mm)	24.38 (2.29)	0.75	25.85 (2.70)	0.84	0.00
MalW (mm)	62.88 (3.73)	0.92	62.16 (3.94)	0.96	0.01
MLATi (deg)	14.01 (3.73)	0.84	14.53 (3.64)	0.83	0.18
TaAL (mm)	33.35 (2.94)	0.75	32.44 (2.75)	0.68	0.00
TaW (mm)	24.60 (2.34)	0.73	26.61 (2.86)	0.85	0.00
SRTa (mm)	22.85 (2.21)	0.58	21.64 (2.90)	0.59	0.00
MLATa (deg)	1.44 (1.16)	0.11	1.59 (1.32)	0.03	0.38

See Table 2 for definitions of the parameters

In terms of inter-rater analysis, good to very good reliability was found for all the parameters, except for poor reliability (ICC = 0.20) for MLATa (Table 4). The senior examiner showed better inter-session reliability than the junior, as indicated by the higher ICC values (Table 4), most of which were larger than 0.92, except for SRTa (ICC = 0.79) and MLATa (ICC = 0.19). For the junior examiner, most of the parameters showed good inter-session reliability (ICC > 0.61), except for MDA (ICC = 0.46) and MLATa (ICC = 042) (Table 4).

**Table 4:** Inter-rater and inter-session reliability of the measurements of ankle morphological parameters in terms of coefficients of variance (CV) and intra-class correlation coefficients (ICC) by the senior and junior examiners

	Inter-session				Inter-rater	
	Senior		Junior			
	ICC	CV	ICC	CV	ICC	CV
TiAL	0.95	0.09	0.80	0.08	0.89	0.09
SRTi	0.94	0.23	0.87	0.18	0.67	0.22
APG	0.99	0.75	0.99	0.75	0.98	0.76
APA	0.99	0.70	0.99	0.73	0.98	0.72
MTiTh	0.98	0.06	0.78	0.08	0.80	0.07
MDA	0.98	0.61	0.46	0.69	0.61	0.64
MDV	0.93	0.29	0.83	0.64	0.84	0.46
TiW	0.94	0.09	0.95	0.09	0.77	0.10
MalW	0.99	0.06	0.98	0.06	0.96	0.06
MLATi	0.95	0.23	0.91	0.22	0.92	0.26
TaAL	0.92	0.08	0.66	0.10	0.89	0.09
TaW	0.92	0.09	0.92	0.09	0.62	0.11
SRTa	0.79	0.10	0.61	0.18	0.80	0.12
MLATa	0.19	0.44	0.42	0.44	0.20	0.82

See Table 2 for definitions of the parameters

Most of the tibial parameters were moderately to highly correlated with the corresponding gold standard values for both examiners, except for TiAL (r = 0.46), SRTi (r = 0.22) and MDV (r = 0.01) measured by the senior examiner, and for SRTi (r = 0.40), MDV (r = 0.22) and MDA (r = 0.16) by the junior examiner. In contrast, all the parameters for the talus showed poor to fair correlations with the corresponding standard values for both examiners (r < 0.45, Table 5). Most of the parameters were significantly different from the standard values (*p* < 0.05), except for SRTi, APG, APA and SRTa (Table 5). Medium to large effect sizes were found between measurements by both examiners and the gold standard for most tibial and talar parameters.

**Table 5:** Validity of the measurements by the senior and junior examiners in terms of *r* values from the Pearson’s correlation analysis and *p* values from paired *t* tests of measurements by each examiner with gold standard values

	Senior					Junior					Standard	
	Mean error (%)	SD error (%)	*r*	*d*	*p*	Mean error (%)	SD error (%)	*r*	*d*	*p*	Mean	SD
TiAL (mm)	−7.74	5.87	0.46	0.99	0.00	−8.50	4.68	0.57	0.84	0.00	29.22	2.50
SRTi (mm)	−5.36	15.66	0.22	0.44	0.09	−5.29	13.89	0.40	0.50	0.10	28.72	6.95
APG (mm)	−2.88	52.57	0.51	0.01	0.70	−1.09	49.73	0.53	0.08	0.96	4.04	2.49
APA (deg)	2.34	49.40	0.51	0.15	0.37	15.28	59.81	0.54	0.20	0.47	7.99	4.78
MTiTh (mm)	−4.08	2.65	0.64	1.97	0.00	−8.26	3.30	0.60	1.03	0.00	44.23	1.93
MDA (mm)	−59.92	16.79	0.58	2.26	0.00	−70.27	14.05	0.16	1.45	0.00	13.67	7.41
MDV (mm)	365.22	450.11	0.01	1.44	0.00	221.06	292.59	0.22	2.89	0.00	2.67	1.61
TiW (mm)	−28.70	3.38	0.61	4.12	0.00	−25.81	4.65	0.52	6.09	0.00	33.68	1.53
MalW (mm)	−0.84	0.92	0.97	0.30	0.04	−1.63	0.80	0.97	0.12	0.00	63.65	3.56
MLATi (deg)	7.25	13.64	0.72	0.64	0.01	16.70	17.33	0.63	0.45	0.00	12.73	3.28
TaAL (mm)	8.28	14.15	0.32	0.58	0.01	6.02	13.86	0.33	0.77	0.04	30.47	4.88
TaW (mm)	16.97	8.71	0.45	2.48	0.00	26.45	11.17	0.41	2.01	0.00	20.48	1.89
SRTa (mm)	7.31	14.97	0.16	0.11	0.31	0.45	12.75	0.14	0.29	0.69	22.13	3.34
MLATa (deg)	210.49	349.58	0.14	0.68	0.05	223.48	367.74	0.12	0.58	0.03	1.02	1.04

Effect sizes (Cohen’s *d*) between the measurements and gold standard values were also calculated. Significance level was set at α = 0.05. See Table 2 for definitions of the parameters. The measurement errors were determined as the differences between the measurements and the corresponding standard values, and represented as percentages of the standard values

**Fig. 6:**
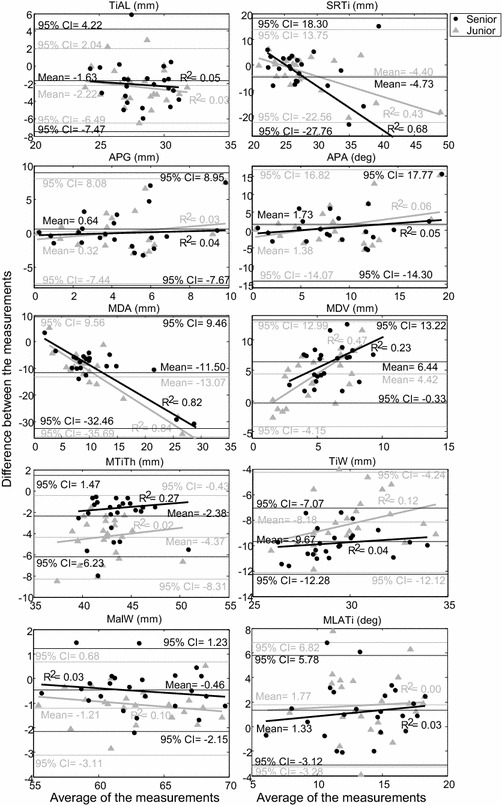
Bland and Altman plot of each morphological parameter. The Bland and Altman plot of every ankle’s morphological parameters measured by each examiner compared to the gold standard, for the tibial bones

**Fig. 7:**
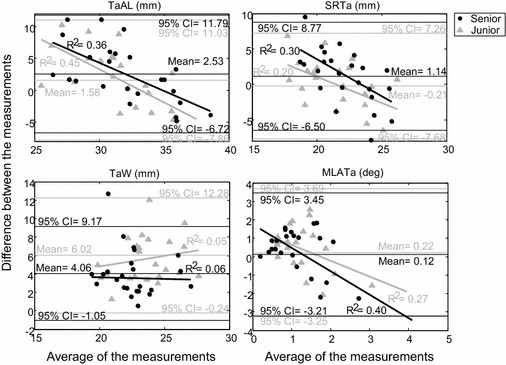
Bland and Altman plot of each morphological parameter. The Bland and Altman plot of every ankle’s morphological parameters measured by each examiner compared to the gold standard, for the talar bones

The Bland and Altman analysis showed that both examiners tended to underestimate most of the tibial parameters and to overestimate most of the talar parameters as compared to gold standard values (Figs. 6, 7). For measurements by the senior examiner, the biases (mean differences) and the 95 % confidence intervals of the differences from the gold standard values were significantly smaller than zero for TiAL, MDA, MTiTh, TiW and MalW, while those for TaAL, TaW, MDV and MLATi showed the opposite (Fig. 6). Similar results were also found for the junior examiner, except that the biases (mean differences) and the 95 % confidence intervals of the differences from the gold standard values were significantly smaller than zero for SRTi but with no difference for TaAL (Fig. 7).

## Discussion

The current study aimed to evaluate the validity and inter-rater, intra-rater, and inter-session reliability of planar radiograph-based measurements of ankle morphological parameters, and to quantify the effects of experience on these measurements for a senior and a junior examiner. Most of the morphological parameters showed good to very good intra-rater, inter-session and inter-rater reliability for both examiners (Tables 3, 4). However, the senior examiner had better inter-session reliability. In terms of accuracy, most of the tibial parameters were moderately to highly correlated to gold standard values, but most talar parameters were found to have poor to fair correlations. The current results may be used as a guideline for future applications in follow-up evaluations of morphology, pre-surgical planning [26, 27] and support for implant design for TAA.

Generally, clinical experience did not appear to affect the intra-rater reliability in the measurement of most ankle parameters on the planar radiographs. Both examiners showed good to very good intra-rater reliability for most of the parameters (Table 3). One exception was the sagittal radius of the tibial mortise (SRTi), for which the senior examiner showed good intra-rater reliability while the junior examiner showed only moderate reliability. This can be explained by the difficulty in identifying the five bony landmarks necessary to define the tibial mortise profile from overlapped images of the distal tibia, fibula and talus on the sagittal radiograph. The corresponding identification of the trochlea tali profile was even more difficult than the tibial mortise because of the small size of the talus, combined with the overlapping of the medial and lateral trochlea. The fact that SRTa and SRTi showed only poor to moderate reliability compared to MLATa should be interpreted carefully because in the sagittal plane the radii of curvature of the tibial and talar components of a TAA have a great impact on the joint’s mobility and stability [28]. Generally, if the radius of curvature of the talar component is smaller than normal, the range of joint motion may increase and the ligaments may become slack, leading to joint laxity. On the other hand, a radius of curvature greater than normal will reduce the range of joint motion and tighten the ligaments, likely resulting in a higher risk of ligament injuries. It appeared that clinical experience helped to identify the bony landmarks reliably for defining the profiles of the tibial mortise from the overlapped images. Such information may be used to guide the selection of the tibial component, which can then be used to find a matching talar component. It is suggested that whenever possible more reliable measurements of SRTi and SRTa by a more experienced examiner should be used to better guide the selection of TAA design and size.

Clinical experience also helped increase the reliability of repeated evaluations such as in a follow-up assessment, as indicated by the higher inter-session reliability of the senior examiner for most of the parameters here analyzed (Table 5). Reliable measurements between sessions before TAA are essential for parameters such as MDA, a crucial parameter for the choice of the optimal level for bone saw cuts. The results of the current study showed that compared to the senior examiner, the junior had reduced inter-session reliability in measuring MDA, MTiTh, TiAL, TaAL and SRTa. These parameters are related to the bone saws or to the choice of the prosthesis size, indicating that care should be exercised by less experienced examiners when measuring these parameters. It is suggested that clinical decisions that require follow-up assessments or repeated anatomical measurements are made by more experienced surgeons, or by less clinically experienced examiners who have had extensive relevant training.

Whereas both senior and junior examiners showed good to very good intra-rater reliability in measuring most morphological parameters at the ankle from planar radiographs, differences in clinical experience appeared to affect the inter-rater reliability considerably. In fact, about half of these parameters were found to have very good inter-rater reliability, but the other half showed only poor to good inter-rater reliability (Table 4). Among those parameters with poor to good inter-rater reliability, statistical differences were also found in the measured values between the examiners for some parameters such as MDA, MTiTh, TiW, TaW and SRTa (Table 3). Therefore, further examination of the validity of these measurements is needed to reveal the effects of individual examiners on the observed between-examiner differences.

The validity of the current measurements by each of the examiners was assessed by comparing these to corresponding gold standard values. Most of the tibial parameters had moderate to excellent correlations with the gold standard values but were underestimated for both examiners, in contrast to most of the talar parameters that were overestimated and had only poor to fair correlations (Table 5). Further examination of the correlations revealed that parameters with a moderate to excellent correlation were primarily those related to measurements of length, while those with poor to fair correlations were mainly related to measurements of angles. The poor correlation in the talar parameters could be attributed to the double dome shape of the talus which produced overlapped images on the planar radiographs. Clinical experience also affected the correlations and differences between the measurements and the gold standard values. For MDA, MTiTh, TiW, TaW and SRTa, which showed poor to good inter-rater reliability, the senior examiner showed higher correlations than the junior (Table 5). In fact, for most of the parameters the former showed smaller differences between the measurements and the gold standard values than the latter (Table 5). The senior also performed better than the junior examiner in terms of inter-session reliability for MDA, MTiTh, and SRTa (Table 5). These results suggest that clinical experience affected the inter-rater and inter-session reliability, and that more clinically experienced examiners have better correlations with and smaller differences from the gold standard values.

Assessing the reliability of ankle morphological measurements and the effects of clinical experience on such relevant reliability has significant clinical consequences. For example, clinical success of TAA nowadays depends heavily on the available information on the morphology of the bones of the ankle, which is critical for the design of the prostheses and the procedures for their surgical implantation [1, 2]. The parameters considered in the current study define the radius, width and length of the ankle bones, whose accurate and reliable measurements are necessary for the image-based diagnosis, treatment planning and outcome assessment for ankle-related disorders. This is critical because in the clinical setting multiple practitioners may be involved and may perform measurements routinely on the radiographs during the course of a patient’s care. Since reliability and reproducibility depend on techniques that minimize variability and maximize accuracy, the effects of clinical experience were thus evaluated in this study.

The current study evaluated the validity and reliability of radiograph-based measurements of ankle morphology from a number of specimens of healthy ankles in the neutral position. However, deformity may be present in ankles with trauma or disease, which may affect the measurement reliability. Further studies are necessary to evaluate these effects. On the other hand, measurements of the ankle morphology on planar radiographs can be sensitive to the positioning of the joint in space during imaging. The current study addressed only the reliability within and between examiners who identified the landmarks on identical planar images of the ankles. However, real-world variability owing to challenges in reliable joint positioning during planar X-ray imaging may be underestimated. Further investigation is needed to identify the effects of ankle positioning on the reliability of the morphological measurements.

## Conclusions

The present study evaluated the validity and inter-rater, intra-rater and inter-session reliability of planar radiograph-based measurements of ankle morphological parameters, and quantified the effects of the examiners’ clinical experience on the reliability and validity of these measurements. While most of the morphological parameters showed good to very good intra-rater, inter-session and inter-rater reliability for both examiners, clinical experience appeared to improve inter-rater and inter-session reliability. Clinical experience helped increase the reliability of repeated evaluations after a longer interval, such as in a follow-up assessment. Most of the tibial parameters had moderate to excellent correlations with the gold standard values but were underestimated by both examiners, in contrast to most of the talar parameters that were overestimated and had only poor to fair correlations. The current results suggest that clinical decisions that required follow-up assessments or repeatable measurements such as for prosthesis sizing or bone saw cuts should be made by more experienced surgeons or by less clinically experienced examiners providing they have had extensive measurement training.

## Abbreviations

2D: two-dimensional
3D: three-dimensional
A/P: anteroposterior
CT: computerized tomography
CV: coefficients of variance
DRR: digitally reconstructed radiographs
ICC: intraclass correlation coefficient
M/L: mediolateral
OA: osteoarthritis
SD: standard deviations
S/I: superoinferior
TAA: total ankle arthroplasty
